# Engineering three-dimensional bone macro-tissues by guided fusion of cell spheroids

**DOI:** 10.3389/fendo.2023.1308604

**Published:** 2023-12-19

**Authors:** Vinothini Prabhakaran, Ferry P.W. Melchels, Lyndsay M. Murray, Jennifer Z. Paxton

**Affiliations:** ^1^Anatomy@Edinburgh, Edinburgh Medical School, Biomedical Sciences, University of Edinburgh, Edinburgh, United Kingdom; ^2^Centre for Discovery Brain Sciences, College of Medicine and Veterinary Medicine, University of Edinburgh, Edinburgh, United Kingdom; ^3^School of Engineering and Physical Sciences, Institute of Biological Chemistry, Biophysics and Bioengineering, Heriot-Watt University, Edinburgh, United Kingdom; ^4^Future Industries Institute, University of South Australia, Adelaide, SA, Australia; ^5^Euan McDonald Centre for Motor Neuron Disease Research, University of Edinburgh, Edinburgh, United Kingdom

**Keywords:** bone, scaffold-free, bioassembly, spheroid, macrotissue, tissue engineering

## Abstract

**Introduction:**

Bioassembly techniques for the application of scaffold-free tissue engineering approaches have evolved in recent years toward producing larger tissue equivalents that structurally and functionally mimic native tissues. This study aims to upscale a 3-dimensional bone *in-vitro* model through bioassembly of differentiated rat osteoblast (dROb) spheroids with the potential to develop and mature into a bone macrotissue.

**Methods:**

dROb spheroids in control and mineralization media at different seeding densities (1 × 10^4^, 5 × 10^4^, and 1 × 10^5^ cells) were assessed for cell proliferation and viability by trypan blue staining, for necrotic core by hematoxylin and eosin staining, and for extracellular calcium by Alizarin red and Von Kossa staining. Then, a novel approach was developed to bioassemble dROb spheroids in pillar array supports using a customized bioassembly system. Pillar array supports were custom-designed and printed using Formlabs Clear Resin^®^ by Formlabs Form2 printer. These supports were used as temporary frameworks for spheroid bioassembly until fusion occurred. Supports were then removed to allow scaffold-free growth and maturation of fused spheroids. Morphological and molecular analyses were performed to understand their structural and functional aspects.

**Results:**

Spheroids of all seeding densities proliferated till day 14, and mineralization began with the cessation of proliferation. Necrotic core size increased over time with increased spheroid size. After the bioassembly of spheroids, the morphological assessment revealed the fusion of spheroids over time into a single macrotissue of more than 2.5 mm in size with mineral formation. Molecular assessment at different time points revealed osteogenic maturation based on the presence of osteocalcin, downregulation of Runx2 (*p* < 0.001), and upregulated alkaline phosphatase (*p* < 0.01).

**Discussion:**

With the novel bioassembly approach used here, 3D bone macrotissues were successfully fabricated which mimicked physiological osteogenesis both morphologically and molecularly. This biofabrication approach has potential applications in bone tissue engineering, contributing to research related to osteoporosis and other recurrent bone ailments.

## 1 Introduction

Bone defects and diseases are prevalent worldwide with high morbidity rates and significant clinical challenges in repair and regeneration. Metabolic, metastatic, and genetic bone diseases cause severe pain, reduced mobility, and increased socioeconomic costs and can also lead to secondary defects like fractures (1). Pharmacological drugs such as antiresorptive agents and osteoanabolics were developed for treating these debilitating diseases, and surgical grafts are also common in orthopedic practice to repair and rebuild damaged bones (2). However, clinical drug trials have limitations such as insufficient trial patients and a greater risk of unpredicted side effects (3, 4). Also, surgical auto- and allografts are in short supply along with other limitations such as donor site morbidity, graft rejection, and infection (5). To minimize these limitations, bone tissue engineering plays a crucial role in developing *in-vitro* biomimetic models for preclinical drug tests (6) and as replacement for bone grafts (7).

Osteoblast monolayer cell cultures are common *in-vitro* models used to investigate physiopathological and pharmacological mechanisms in bone diseases as well as toxicity tests of investigative drugs. However, cellular and extracellular matrix (ECM) interactions in monolayer cell cultures are not biomimetic due to their two-dimensional nature (8). Alternatively, three-dimensional (3D) cultures recapitulate the complex cellular microenvironment more closely related to natural bone tissues (8). Different scaffold materials have been used for 3D cultures which either act like native ECM allowing growth and differentiation of cells, e.g., decellularized ECM (9), or provide an environment for cells to produce their own ECM, e.g., functional hydrogels (8). Despite the interest in scaffolding materials for bone tissue engineering, there are significant limitations, specifically the high costs, complex fabricating procedures, limited cell density, hindrance to mechanotransduction between cells, and fate of the foreign material after implantation for applications in regenerative medicine (10). Thus, scaffold-free tissue engineering is gaining importance in developing clinically useful tissue constructs by excluding the use of exogenous scaffolds (11).

Scaffold-free 3D models, especially spheroids, have great potential in fabricating biomimetic tissues due to their self-assembling and self-organizing properties which better reflect natural tissues. This approach has varied applications as drug screening models, developmental and disease models, and large-scale biofabricated tissue to replace irreversibly damaged tissues (12). In recent years, spheroids (i.e., microtissues) have been considered as building blocks to fabricate macrotissues and organs through guided assembly and fusion (13–15).

Three-dimensional spheroid-based bioassembly approaches are emerging to manufacture large-scale tissues. An automated bioassembly system has been developed by the Woodfield group to produce scaffold-based chondrocyte tissue constructs using a PEGT/PBT copolymer (13). Alternatively, in order to develop a “scaffold-free” osteogenic macrotissue, Heo et al. (16) employed sacrificial materials, i.e., sodium alginate cross-linked with calcium chloride which was removed by citrate after spheroid fusion, making the construct scaffold-free. However, the effect of citrate chelation on calcium of osteogenic tissue was not addressed. Another research group has developed the Kenzan method to form scaffold-free tissues by inserting microneedles into spheroids (17). Although this method has been effective, there is a high possibility of tissue disintegration during the removal process (18). These drawbacks demand an alternative approach to bioassemble osteoblast spheroids into macrotissues without any destructive effects.

Our study aims to fabricate a biomimetic rat osteoblast macrotissue using a customized bioassembly system. To achieve this aim, osteogenic induction using mineralization media was first studied to observe cell proliferation, cellular arrangement, and extracellular matrix synthesis in osteoblast spheroids. Spheroids were then bioassembled and assessed to demonstrate the biomimetic nature of the fabricated macrotissue construct by morphological and molecular analyses.

## 2 Materials and methods

### 2.1 Cells

Rat osteoblasts (RObs) were procured from Cell Applications, Inc. (USA) and cultured according to the manufacturer’s protocol for expansion and differentiation, resulting in a population of differentiated rat osteoblasts (dRObs) that were cryopreserved and thawed when required. dRObs passage numbers 5 to 12 were used in this study.

### 2.2 Culture media

#### 2.2.1 Growth media

Dulbecco’s modified Eagle’s medium (DMEM) containing high glucose with sodium pyruvate and L-glutamine (Product #41966052, Gibco™, Fisher Scientific, UK) was supplemented with 10% fetal bovine serum (FBS; Product #FB-1001, LabTech Inc., UK) and 1% antibiotic–antimycotic solution (ABAM; Product #A5955, Sigma-Aldrich, UK). This supplemented DMEM was used as a standard growth medium (GM) for cell culture.

#### 2.2.2 Mineralization media

Mineralization media (MM) was prepared by further supplementing GM with 10 nM of dexamethasone (Product #D4902, Sigma-Aldrich, Germany), 10 mM of β-glycerophosphate disodium salt (Product #G9422, Sigma-Aldrich, USA), and 10 ng/ml of recombinant human BMP-4 (Product #AF-120-05ET, Peprotech®, UK). L-Ascorbic acid 2-phosphate sesquimagnesium salt hydrate (50 µg/ml) (Product #A8960, Sigma-Aldrich, USA) was freshly added on the day of media usage. The prepared media was filter-sterilized (0.22 µm pore size) before use.

### 2.3 Spheroids’ growth and mineralization

The dRObs were plated in triplicates for spheroid formation in 96-well “U” bottom cell-repellent plates (Product #650970, CELLSTAR^®^, Greiner Bio-One, UK) at three different cell seeding densities, i.e., 1 × 10^4^, 5 × 10^4^, and 1 × 10^5^ cells/150 µl of GM per well, and incubated at 37°C and 5% CO_2_. After 48 h, the GM was replaced with MM, while control spheroids were maintained in GM. The spheroids were assessed for cell proliferation and viability, presence of a necrotic core, and ECM calcium deposits on days 7, 14, 21, and 28.

#### 2.3.1 Cell proliferation and viability

The dROb spheroids were dissociated by placing them in 100 µl of accutase (Product #00-4555-56, Invitrogen™ Thermo Scientific, CA, USA) and incubating for 40 min at 37°C and 5% CO_2_. Cell count and viability were assessed by a trypan blue staining method (Product #15250-061, Thermo Fisher, USA) according to the manufacturer’s protocol. One-way ANOVA and *post-hoc* Tukey test were performed to compare cell proliferation and viability among different seeding densities (*N* = 3). The spheroid diameter was measured by Fiji/ImageJ software using images taken on Leica DMi1 phase contrast inverted microscope.

#### 2.3.2 Necrotic core assessment

Spheroids were washed with phosphate buffered saline (PBS 1×) twice and fixed with 4% paraformaldehyde (Product #J19943-K2, Thermo Scientific, Belgium) for 1 h at room temperature. As each of the spheroids is of an extremely small size, they were embedded in agarose blocks before wax processing. In brief, a drop of 2% agarose (Product #15510-027, Invitrogen, UK) was placed on a glass slide onto which a spheroid was deposited and covered with another drop of 2% agarose. After trimming the agarose blocks into a cubic shape, they were wax-processed using a Leica ASP300S tissue processor and embedded in paraffin wax blocks. Paraffin-embedded spheroids were sliced into 10 µm sections using a rotary microtome (Leica Biosystems, UK) and placed onto SuperFrost Plus™ glass slides. Harris hematoxylin and eosin staining (H&E) was performed on spheroid sections according to the manufacturer’s protocol. In brief, the sections were dewaxed in xylene, hydrated with alcohol series (100%, 90%, 70%, and running water), followed by hematoxylin (Product #RBA-4205-00A, CellPath, UK) for 3 min which was differentiated by acid alcohol and eosin staining (Product #6766008, Shandon™, Fisher Scientific, UK) for 2 min which was differentiated by potassium alum and final dehydration by alcohol series, cleared by xylene, and mounted with DPX using coverslips.

#### 2.3.3 Extracellular matrix production (calcium deposits)

Fixed spheroids were subjected to Alizarin red staining for assessing calcium deposits. First, the spheroids were washed twice with distilled water, and 100 µl of Alizarin red stain (Product #2003999, EMD Millipore, USA) was added and incubated at room temperature and protected from light for 10 min. Then, spheroids were thoroughly washed four times with distilled water and observed for calcium deposits under Leica DMi1 inverted microscope (bright field).

### 2.4 Bioassembly of spheroids

#### 2.4.1 Three-dimensional modeling and printing

**Figure 1:**
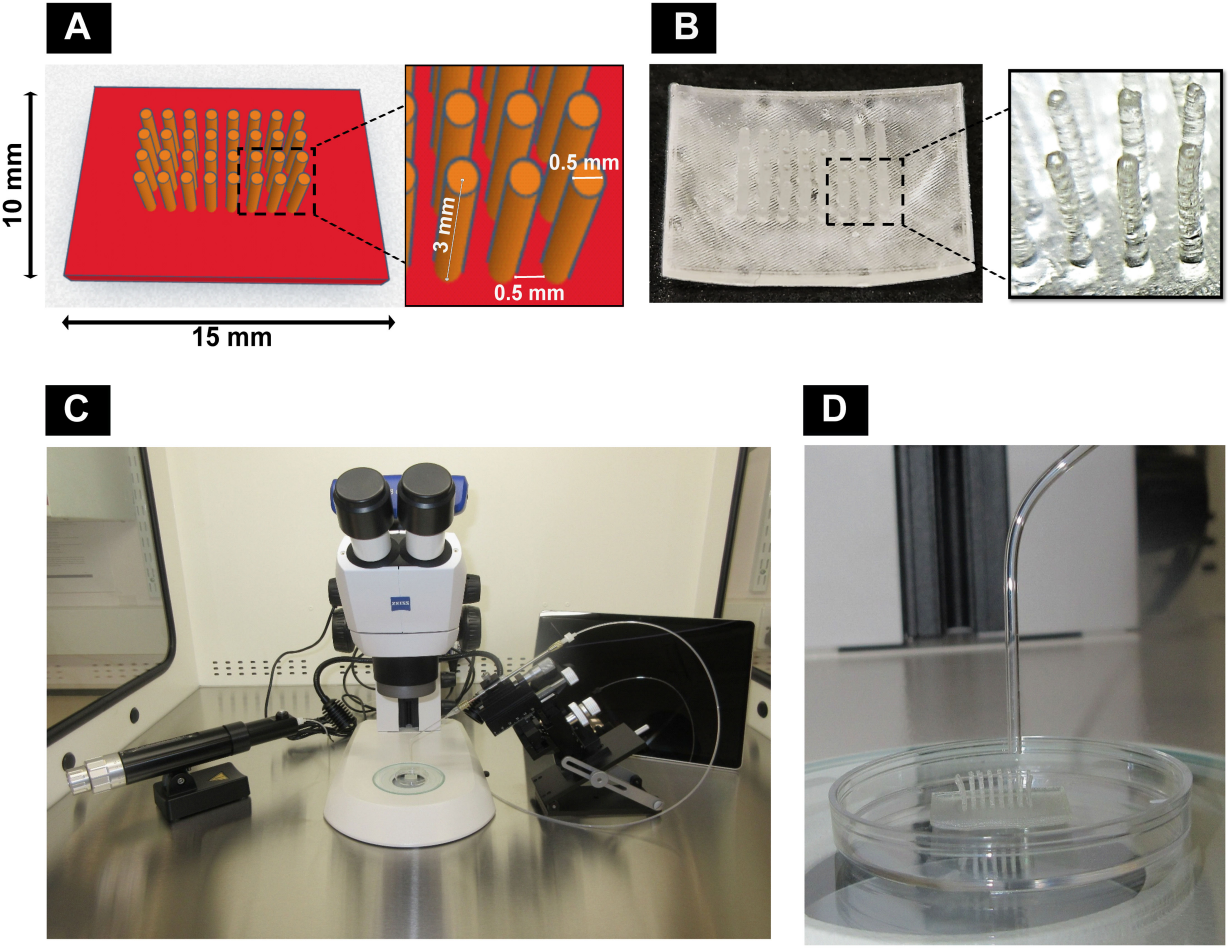
Components of the customized bioassembly system: **(A)** computer-aided design with dimensions using Tinkercad and **(B)** 3D-printed pillar array supports using Formlabs Clear Resin^®^ with Formlabs Form2 3D printer. **(C)** Customized spheroid bioassembly system with CellTram^®^4r Air, micromanipulator, stereomicroscope, and Microsoft Surface Pro 9 installed with Zeiss Labscope. **(D)** Pillar array support along with bent capillary to deposit spheroids.

The pillar array support used in this study was designed with an online 3D computer-aided design (CAD) program (https://www.tinkercad.com/) with 0.5 mm pillar-to-pillar distance, 0.5 mm pillar diameter, 3 mm pillar height, and 1 mm base thickness (15 mm L × 10 mm W) (**Figure 1A**). The designs were printed (**Figure 1B**) with “Formlabs Clear Resin^®^” (Product #RS-F2-GPCL-04) using a Formlabs Form2 3D printer and postprocessed by rinsing in isopropyl alcohol (IPA) followed by postcuring for 60 min within a UV cabinet (UVP CL-1000L, 365 nm, 3 mW/cm^2^). Prints were then extracted in IPA within a Soxhlet apparatus overnight. Before use, the printed materials were sterilized by 70% alcohol for 30 min followed by a PBS (1×) wash. Based on preliminary studies (data not included), the postprocessed resin material used in this study has been confirmed as non-cytotoxic.

#### 2.4.2 Customized 3D bioassembly system

A customized setup (**Figure 1C**) was developed by assembling a stereomicroscope (Stemi305, Zeiss, UK) with a camera connected to a Zeiss Labscope software in Microsoft Surface Pro 9 (Product #QCH-00003, XMA Ltd., UK) and a manual micromanipulator (Product #M3301-M3-R, World Precision Instruments, UK) to fix a capillary holder connected to CellTram^®^4r Air (Product #5196000013, Eppendorf, UK), inside a vertical laminar flow hood (Product #VLF-36, Purair^®^, UK). Borosilicate capillaries (Product #PG52151-4, WPI, UK) of the size 1.5 mm OD were heat bent slightly (**Figure 1D**) and inserted into the capillary holder.

#### 2.4.3 Three-dimensional bioassembly of bone cell spheroids

Fifteen-day-old GM and MM cultured dROb spheroids were aspirated into the capillary and transferred to the sterile pillar array supports under microscopic guidance. Six spheroids were deposited in between pillars in a single-layered fashion and incubated at 37°C and 5% CO_2_ in GM and MM, respectively (*N* = 3). Spheroids were removed from supports on days 2, 4, and 6 to determine the extent of fusion. In brief, pillars were cut using a surgical blade (size 22), and spheroids were carefully manipulated to detach from the base using a 22G needle and by inverse tapping into media. The removed spheroids were cultured (*vide infra*) or fixed with 4% paraformaldehyde for 2 h. H&E staining was performed on wax-embedded sections (10 μm).

#### 2.4.4 Scaffold-free culture of bioassembled 3D macrotissues

After the successful removal from the pillar array supports, the fused spheroids (macrotissue) were cultured in a 24-well cell-repellent plate (Product #662970, CELLSTAR^®^, Greiner Bio-One, UK) at 37°C and 5% CO_2_ (*N* = 3) in 1 ml of mineralization media to observe further fusion changes in scaffold-free conditions on days 2, 4, and 8 after removal (depicted as dAR2, dAR4, and dAR8, where dAR is “day after removal”). Media was changed every 2–3 days.

### 2.5 Morphological assessment

#### 2.5.1 Histological staining

Scaffold-free cultured macrotissues over time from dAR2 to dAR8 were fixed with 4% paraformaldehyde for 2 h and sectioned at 10 μm thickness after wax embedding. H&E, Alizarin red, and Von Kossa staining were performed according to the manufacturer’s protocol.

*Alizarin red staining*: After dewaxing and hydrating, the sections were covered with Alizarin red solution (product #2003999, EMD Millipore) for 5–15 min followed by blotting with filter paper and dehydrating by acetone and acetone–xylene mix (1:1), cleared by xylene, and mounted with DPX.

*Von Kossa staining* (Product #ab150687, Abcam, UK): After dewaxing and hydrating, the sections were incubated with 5% silver nitrate under UV light for 1 h, followed by 5% sodium thiosulfate for 2–3 min at room temperature and nuclear fast red for 5 min. The sections were thoroughly washed with distilled water between each step, then dehydrated with absolute alcohol, cleared with xylene, and mounted with DPX.

#### 2.5.2 Scanning electron microscopy

Scanning electron microscopic imaging was performed on dAR8 fused spheroid macrotissue (i.e., day 25 from the initial seeding date) cultured in mineralization media and compared with approximately similar-aged spheroid in growth media (day 28). They were fixed with 4% paraformaldehyde and stored in PBS 1× at 4°C until SEM sample preparation. In brief, the samples were fixed in a solution of 3% glutaraldehyde in 0.1 M of sodium cacodylate buffer (pH 7.3) for 2 h. They were then washed in 3 × 10-min changes of 0.1 M sodium cacodylate buffer. Samples were then postfixed in 1% osmium tetroxide in 0.1 M of sodium cacodylate buffer for 45 min. A further 3 × 10-min washes were performed in 0.1 M of sodium cacodylate buffer. Dehydration in graded concentrations of acetone (50%, 70%, 90%, and 3 × 100%) for 10 min each was followed by critical point drying using liquid carbon dioxide. After mounting on aluminum stubs with carbon tabs attached, the specimens were coated with 9 nm palladium using a Safematic CCU-010 HV sputter coater. The samples were imaged using a Zeiss Crossbeam 550 at 2 and 7 kV using a probe current of 100 pA. An In-lens detector was used to image surface topography.

### 2.6 Molecular assessment

#### 2.6.1 Gene expression by qRT-PCR

RNA from dAR2 and dAR8 macrotissue was extracted using RNeasy^®^ minikit (Product #74104, Qiagen, USA). RNA from the dROb monolayer in GM on day 7 was used as a control/calibrator. The concentration and purity of RNA samples were evaluated using a NanoDrop spectrophotometer. After quality checking, cDNA synthesis was performed using an RT^2^ first-strand kit (Product #330404, #79254, Qiagen, USA). KAPA SYBR^®^ Fast qPCR universal kit (Product #KK4601, KAPA Biosystems Inc., USA) was used to evaluate gene expression in the samples at an annealing temperature of 58.6°C for 40 cycles in Bio-Rad CFX Connect Real-Time PCR Detection System. Target genes were alkaline phosphatase (ALP) [forward primer (f): 5`-GACCCTGCCTTACCAACTC-3`, reverse primer (r): 5`-CCCATACCATCTCCCAGGAA-3`] and Runx2 (f: 5`-GCTTCTCCAACCCACGAATG-3`, r: 5`-GAACTGATAGGACGCTGACGA-3`), and the reference genes were GAPDH (f: 5`-TGTTCTAGAGACAGCCGCAT-3`, r: 5`-GTAACCAGGCGTCCGATACG-3`) and β-actin (f: 5`-TCTGTGTGGATTGGTGGCTCTA-3`, r: 5`-AGGGTGTAAAACGCAGCTCA-3`) (forward and reverse primers from Sigma Aldrich, UK). The amplification was performed in triplicates, and data were analyzed for relative expression using the 2^−▵▵Ct^ method (19).

#### 2.6.2 Osteocalcin immunostaining

Osteocalcin immunostaining was performed on paraffin-embedded bone macrotissues (dAR2, 4, and 8) and control dROb spheroid (day 7 in growth media). Antigen retrieval was performed using citrate buffer (pH 6.0) for 20 min. Sections were blocked using 10% bovine serum albumin (Product #A4503, Sigma Aldrich, USA) and incubated overnight at 4°C with rabbit anti-rat osteocalcin polyclonal antibody (product #PA5-78871, Invitrogen) with a dilution of 1 µg/ml, followed by Alexa Fluor™ 488 donkey anti-rabbit IgG (1:500 dilution) (Product #A21206, Invitrogen, USA) incubation in the dark for 2 h. DAPI-counterstained sections (1:1,000) were imaged using a fluorescent microscope (Leica THUNDER).

### 2.7 Statistical analysis

Data were graphically presented as mean ± standard error in spheroid diameter, cell proliferation and viability, and mean ± standard deviation in relative gene expression analysis. One-way ANOVA and *post-hoc* Tukey test were performed to compare between groups using Past 4.13 software (20).

## 3 Results

### 3.1 Cell proliferation and viability

**Figure 2:**
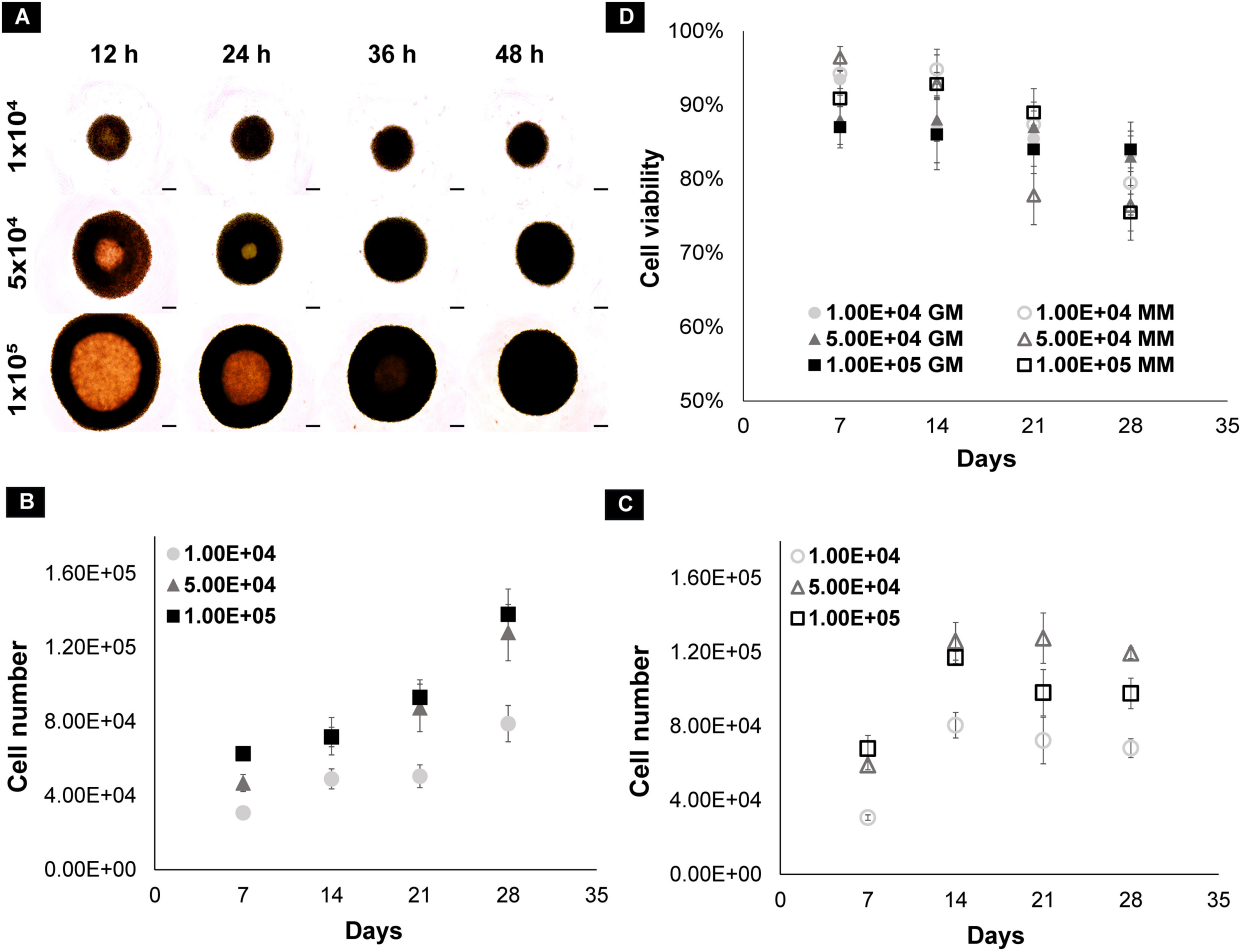
**(A)** Spheroid formation of dROb cells up to 48 h; scale bar: 200 µm. Graphs representing the cell count of dROb spheroids in **(B)** growth media and **(C)** mineralization media and **(D)** cell viability at 1 × 10^4^, 5 × 10^4^, and 1 × 10^5^ seeding densities from day 7 to day 28 (*N* = 3). Legends: “GM” denotes growth media and “MM” denotes mineralization media. Error bars: standard error; *N*: experimental replicates.

dRObs at three seeding densities (1 × 10^4^, 5 × 10^4^, 1 × 10^5^ cells) compactly aggregated at 24 h, 36 h, and 48 h, respectively (**Figure 2A**), and spheroid growth commenced thereafter. In GM, the cell number of all spheroids increased gradually from day 7 to day 28 (**Figure 2B**). In MM, the cell number drastically increased up to day 14 and declined thereafter (**Figure 2C**). Among the three seeding densities, 1 × 10^5^ cell spheroids had a greater reduction in cell number after day 14 in MM. In both GM and MM, cell viability declined with increasing culture time point from day 7 to day 28 in all seeding densities (**Figure 2D**). There was no significant difference (*p* > 0.05) in cell viability between seeding densities at any time point (one-way ANOVA, Tukey *post-hoc* test, *N* = 3).

### 3.2 Spheroid diameter

**Figure 3:**
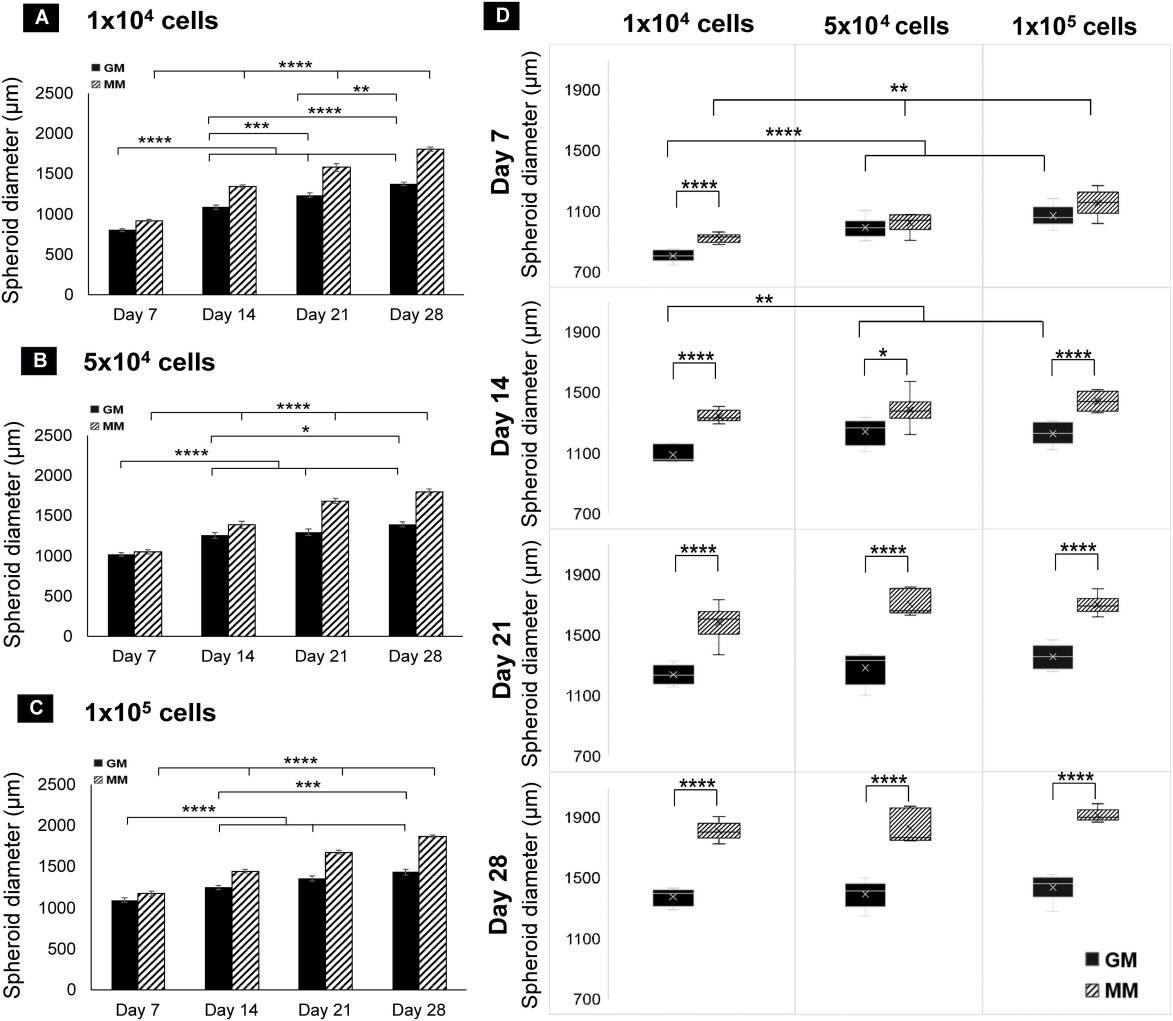
Bar charts of dROb spheroid diameters showing significant differences “between days” at 1 × 10^4^ **(A)**, 5 × 10^4^ **(B)**, and 1 × 10^5^ **(C)** seeding densities (*N* = 3). **(D)** Box plots of spheroid diameter showing significant differences “between media” and “between seeding densities” from day 7 to day 28 (*N* = 3). Legends: “GM” denotes growth media and “MM” denotes mineralization media. Error bars: standard error; significant differences calculated by one-way ANOVA and Tukey *post-hoc* test; *p ≤ 0.05, **p ≤ 0.01, ***p ≤ 0.001, ****p ≤ 0.0001. *N:* experimental replicates.

In all three seeding densities, the diameter of the spheroids increased from day 1 to day 28 in both GM and MM with a significant difference between each time point on days 7, 14, 21, and 28 (**Figures 3A–C**). Spheroids in MM have significantly larger diameters than GM (*p* ≤ 0.01) at all time points and seeding densities except on day 7 in 5 × 10^4^ and 1 × 10^5^ seeded spheroids. Comparing spheroids among the seeding densities, spheroid size was significantly different on days 7 and 14 in GM (*p* ≤ 0.01) and on day 7 only in MM (*p* ≤ 0.01). They reached an approximately similar diameter range (~1.2 to 1.3 mm) on day 21 in GM and on day 14 in MM (no significant difference *p* > 0.05) and increased at a similar rate over time till day 28. Using a low seeding density (1 × 10^4^), a faster increase in spheroid size was observed, and upon reaching a critical size (1.2–1.3 mm), they increased at an equal rate as that of higher seeding densities (**Figure 3D**).

### 3.3 Necrotic core observation

**Figure 4:**
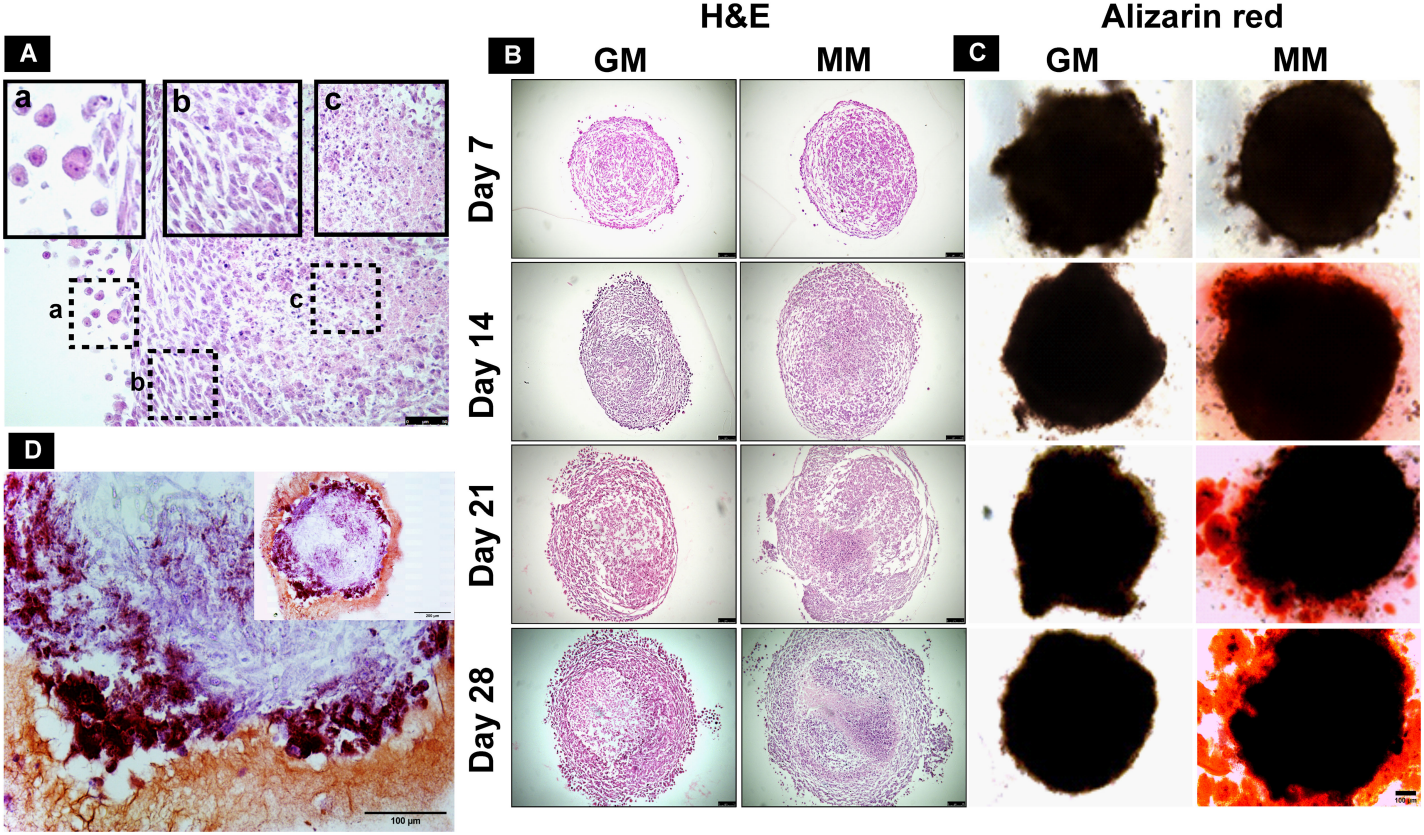
**(A)** H&E-stained section of mineralized dROb spheroid demonstrating different zones: (a) proliferating zone, (b) quiescent zone, and (c) necrotic zone (scale bar: 50 µm). Representative microscopic images of **(B)** H&E-stained spheroid sections (scale bar: 100 µm) and **(C)** Alizarin red-stained spheroids (scale bar: 100 µm) in control (GM) and mineralization media (MM) from day 7 to day 28 (seeding density: 1 × 10^5^cells). **(D)** Section of Alizarin red-stained spheroid (cultured in mineralization media) showing brick red-colored calcium deposits (scale bar: 100 µm); inset image: spheroid section at lower magnification (scale bar: 200 µm).

Cellular arrangement and necrotic cores were observed by H&E staining on spheroid sections (10 µm). The dROb cells were evenly distributed in all spheroids with three typical zones: proliferative, quiescent, and necrotic (**Figure 4A**). The presence of a pink core region with pyknotic, karyorrhectic, and karyolyzed nuclei indicates necrosis (21). The necrotic core size was dependent on the size of the spheroid, i.e., the larger the spheroid size, the greater the necrosis. Any dROb spheroid of more than 1,300 µm demonstrated a necrotic core that continued to widen over time (**Figure 4B**) regardless of the seeding density and media conditions. The presence of a necrotic core was corroborated by cell viability analyses which demonstrated a decline in cell viability over time from day 7 to day 28 (**Figure 2D**).

### 3.4 Extracellular matrix calcium deposits

Alizarin red staining demonstrated the presence of red calcium deposits in spheroids cultured in MM from day 14 in all three seeding densities (**Figure 4C**). Spheroids sectioned after staining revealed brick red-colored calcium deposits (**Figure 4D**). Spheroids in GM do not show the presence of calcium deposits. This suggests that MM induces dROb spheroids to produce an extracellular matrix containing calcium phosphate deposits between day 7 and day 14.

### 3.5 Three-dimensional bioassembly of spheroids

**Figure 5:**
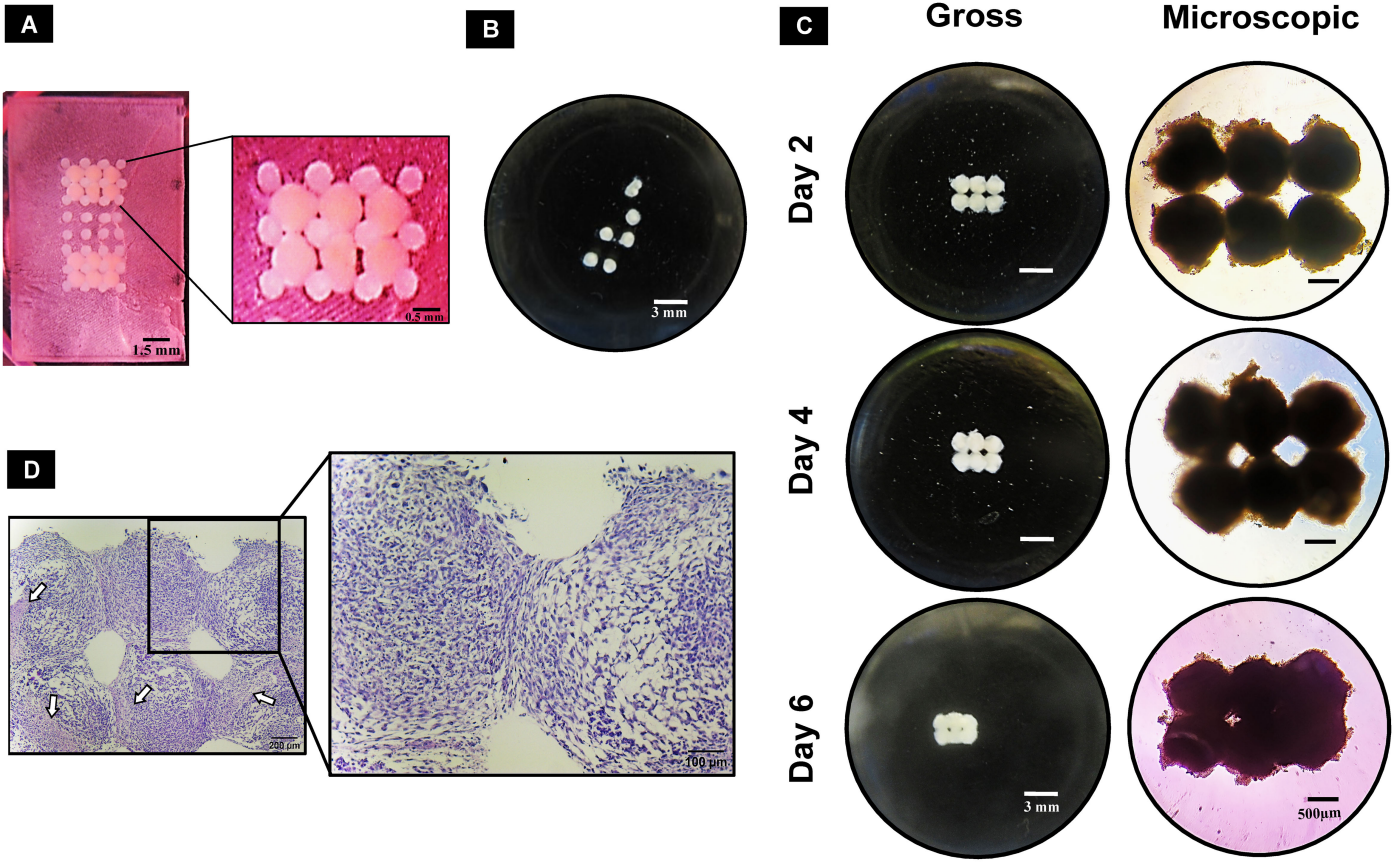
Guided fusion of dROb spheroids: **(A)** dROb spheroids directly after deposition in pillar array support using the customized bioassembly system. **(B)** Gross image of dROb spheroids in growth media (GM) showing no fusion after removal from the pillar array support (scale bar: 3 mm). **(C)** Gross (scale bar: 3 mm) and microscopic images (scale bar: 500 µm) of fused spheroids in mineralization media (MM) removed from the pillar array supports on day 2, day 4, and day 6. **(D)** H&E-stained fused spheroids (removed on day 2) showing tight aggregation between spheroids (scale bar: 200 µm; zoomed image: 100 µm); white arrows indicate necrotic regions.

The 3D-printed pillar array supports had upright pillars to hold the spheroids during the bioassembly process and subsequent culture period until fusion of the spheroids occurred (**Figure 5A**). Removal of spheroids from the pillar array supports on different days (days 2, 4, and 6) revealed that spheroids fused together in MM but not in GM (**Figure 5B**). On day 2 in MM, the spheroids removed from the pillar array supports were clearly fused in regions other than the pillar area (**Figure 5C**). Over time (on days 4 and 6), spheroids were closely connected to each other (**Figure 5C**). However, the removal process was difficult in tightly fused spheroids, as in some cases the pillars remained attached to the spheroids. Considering the difficulty of removal, day 2 after deposition was deduced as the ideal time for spheroid removal from the pillar array supports. H&E staining revealed that the edges of the spheroids were fused compactly (**Figure 5D**). Necrotic core regions were noted to increase over time but did not appear to affect the fusion.

### 3.6 Scaffold-free culture of 3D macrotissue

#### 3.6.1 Fusion and mineralization of macrotissue cultures

**Figure 6:**
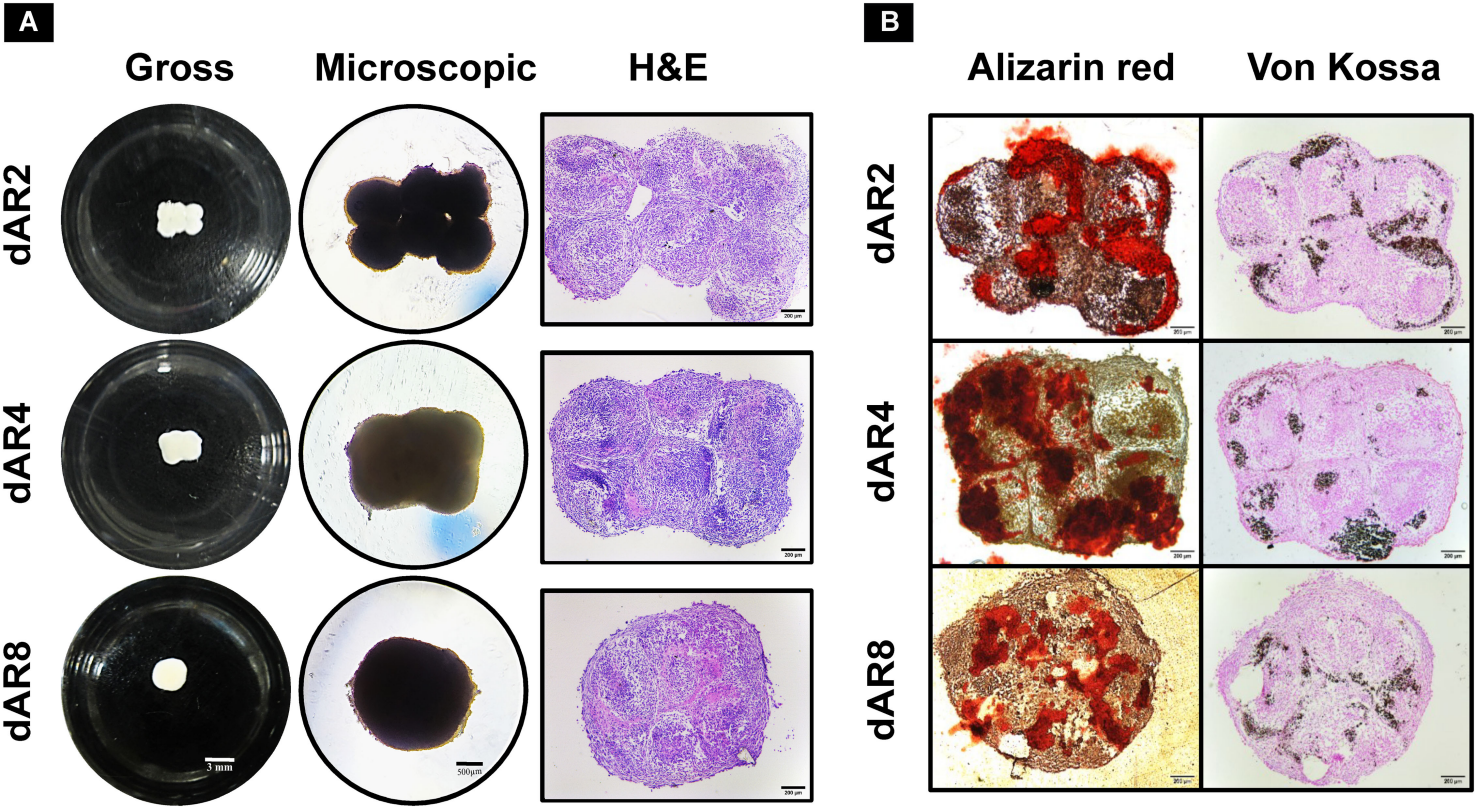
Scaffold-free culture of fused spheroids in mineralization media (MM) on day 2, day 4, and day 8 after removal (depicted as dAR2, dAR4, and dAR8, respectively) from the pillar array supports (*N* = 3). **(A)** Gross (scale bar: 3 mm), microscopic (scale bar: 500 µm), and H&E-stained images (scale bar: 200 µm) of fused spheroids. **(B)** Calcium deposits in Alizarin red and Von Kossa-stained sections of fused spheroids (scale bar: 200 µm).

The removed spheroids cultured in a 24-well cell-repellent plate fused together into macrotissues over time (**Figure 6A**), i.e., 2.64 ± 0.23 mm diameter on dAR8. H&E staining showed the merging of spheroids into one another with an even distribution of cells. Spheroids can be individually identified with a fusion line present in between each until dAR4. On dAR8, the fusion lines disappeared and merged into a single macrotissue with a minimal necrotic core (**Figure 6A**). Alizarin red and Von Kossa staining showed red- and black-stained calcium deposits, respectively, in macrotissues at all time points (**Figure 6B**).

##### 3.6.1.1 Bone-specific hydroxyapatite mineralization in macrotissues

**Figure 7:**
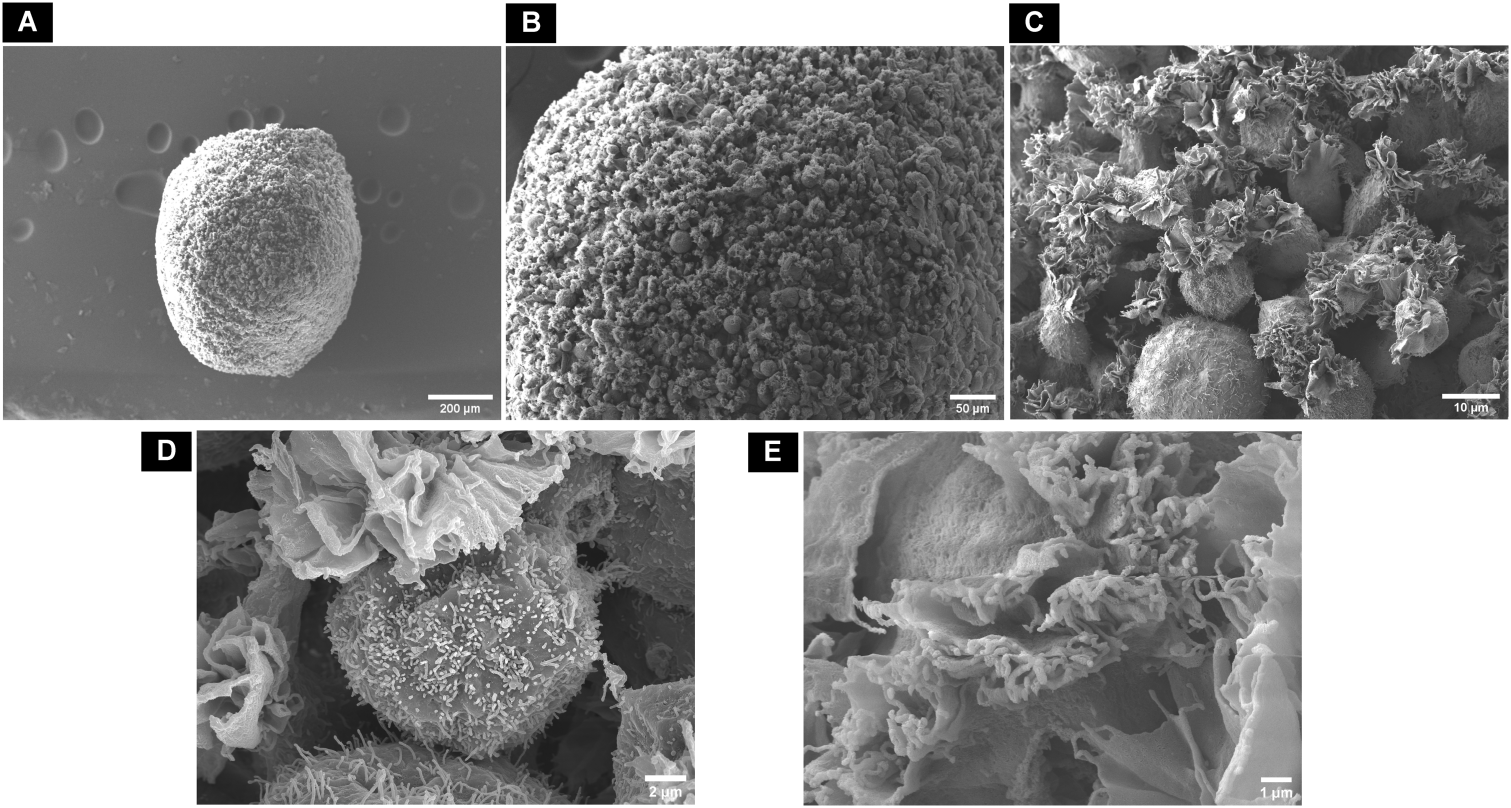
Scanning electron microscopic surface morphology of **(A)** control dROb spheroid on day 28 showing **(B, C)** loosely attached round-shaped cells with **(D, E)** lamellipodia and filopodia.

In control samples (dROb spheroids cultured in GM), the cells were round-shaped and loosely located with no compact cell–cell attachment (**Figures 7A–C**). Lamellipodia (flat ruffled structures) and filopodia (thin filamentous structures) were observed on the surface of each cell; however, the filaments showed minimal contact with adjacent cells (**Figures 7D, E**).

**Figure 8:**
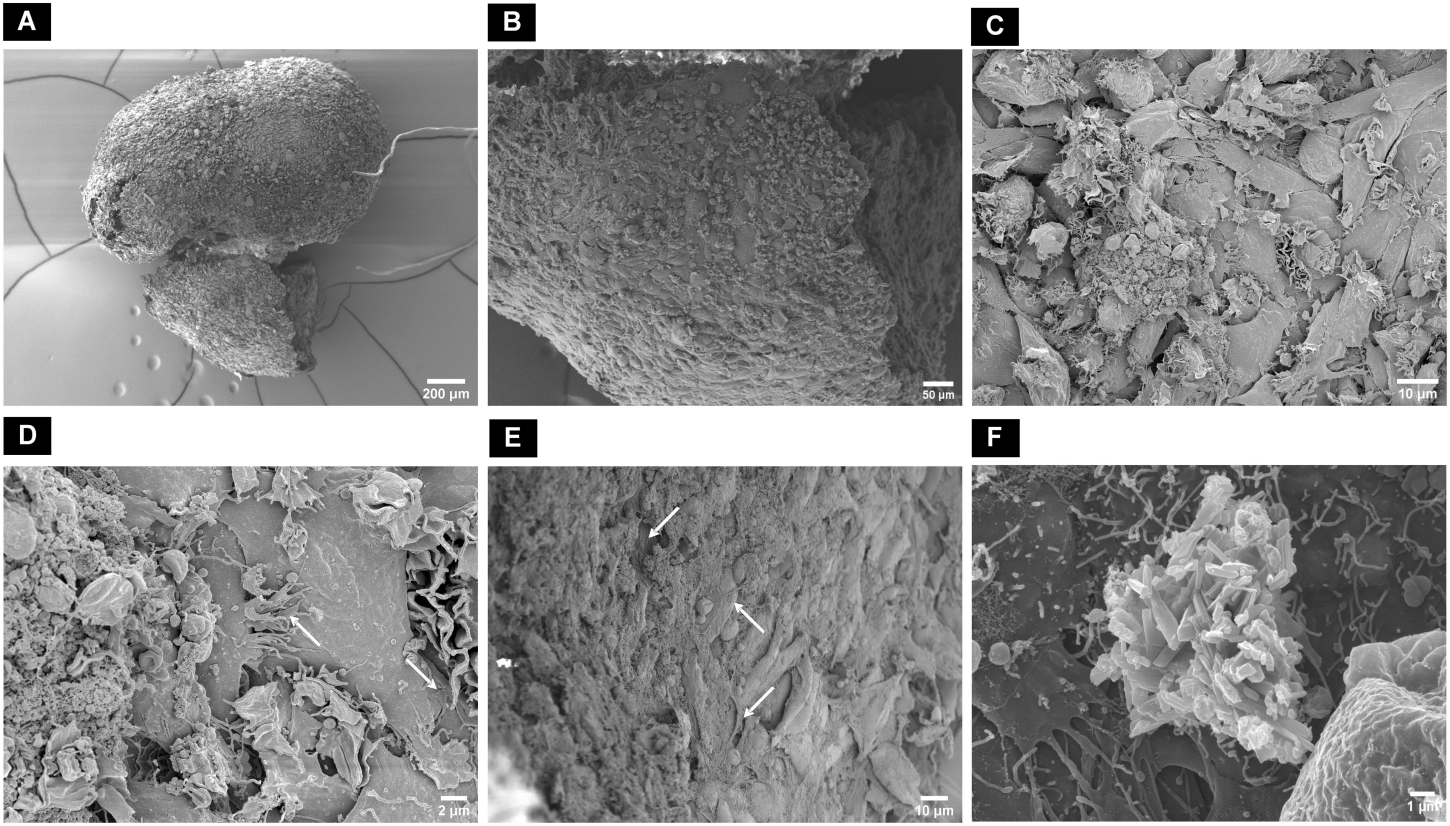
Scanning electron microscopic surface morphology of **(A)** dAR8 macrotissue showing **(B, C)** compactly attached flattened and elongated cells. **(D)** Lamellipodia and filopodia closely attached to adjacent cells (white arrows). **(E)** Fibrous mesh network indicative of collagen fibers (white arrows) with tightly arranged cells in the internal regions of the macrotissue. **(F)** Rod-shaped hydroxyapatite crystals on the surface of macrotissue.

In contrast, in macrotissues cultured in MM, the cells were flattened and elongated with close contact with each other through visible lamellipodia and filopodia (**Figures 8A–D**). A fibrous collagenous network was observed inside the macrotissue with closely packed cells (**Figure 8E**). Rod-shaped crystal structures indicative of hydroxyapatite were observed on the surface of the macrotissue (**Figure 8F**).

#### 3.6.2 Osteogenic differentiation of macrotissues

##### 3.6.2.1 Gene expression

**Figure 9:**
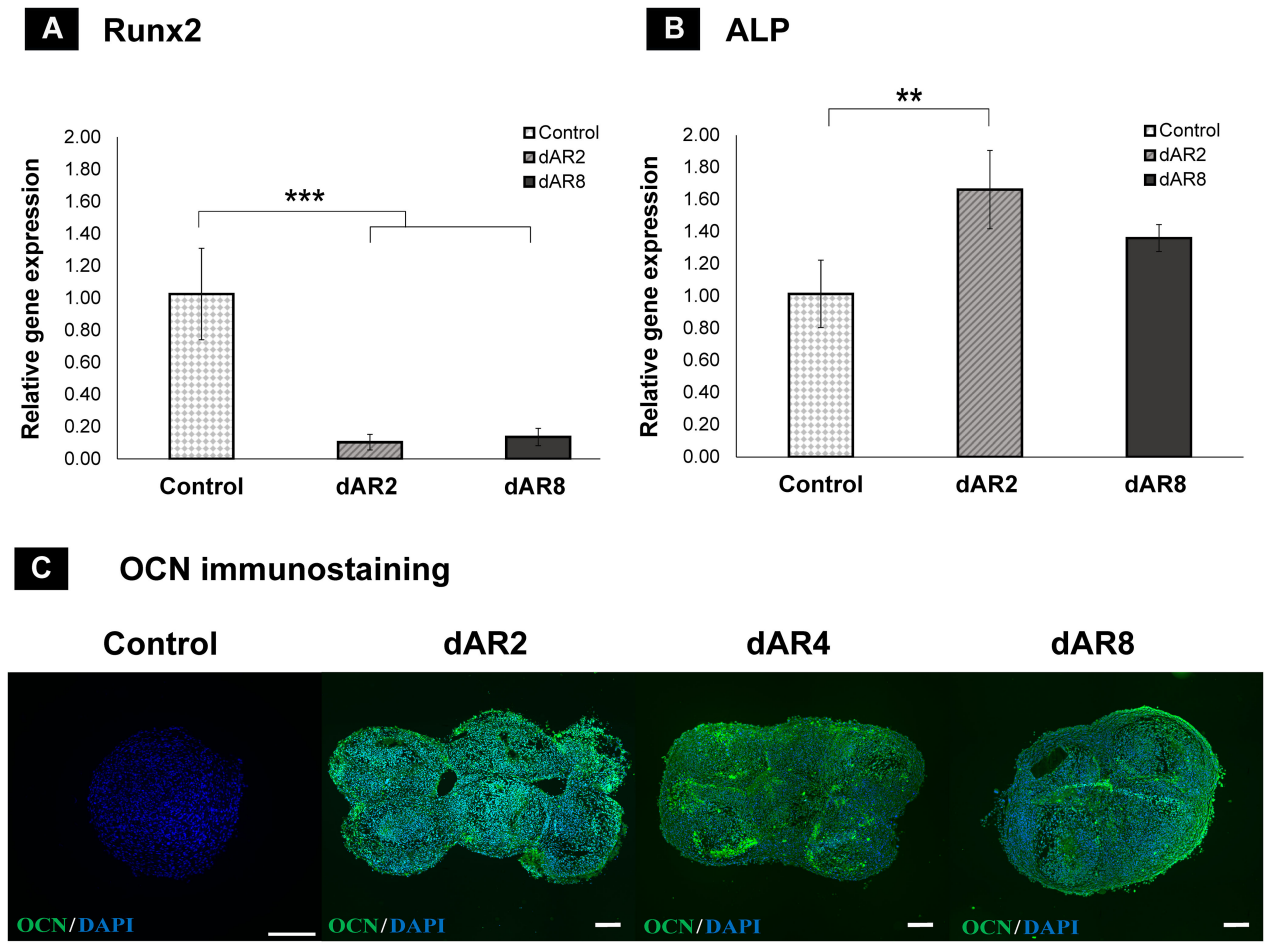
Relative gene expression of **(A)** Runx2 and **(B)** ALP by the 2^−▵▵Ct^ method; Control: day 7 dROb monolayer; dAR2 and dAR8: day 2 and day 8 scaffold-free macrotissue; error bars: standard deviation; significant difference calculated by one-way ANOVA and Tukey *post-hoc* test; **p < 0.01, ***p < 0.001. **(C)** Osteocalcin (OCN) immunostaining (green) on control, day 2, day 4, and day 8 scaffold-free macrotissue with DAPI counterstain (cell nuclei in blue); scale bar: 200 µm.

Runx2 is a transcription factor of early osteoblast differentiation. In osteoblastogenesis, the expression of Runx2 peaks in immature osteoblasts and decreases in mature osteoblasts. The expression of Runx2 on dAR2 and dAR8 significantly downregulated (*p* < 0.001) than the control samples (dRObs monolayer in GM on day 7) (**Figure 9A**). ALP expression continues to increase during bone maturation and mineralization and reduces during terminal osteocyte formation. ALP expression was significantly higher (*p* < 0.01) on dAR2 compared with control, while it was decreased on dAR8, but no statistically significant difference was observed (**Figure 9B**).

##### 3.6.2.2 Osteocalcin immunofluorescence

Osteocalcin is a late differentiation marker expressed by mature osteoblasts and early osteocytes. Immunofluorescence staining confirmed the presence of an osteocalcin marker on dAR2, 4, and 8, whereas the control spheroid lacked the osteocalcin marker (**Figure 9C**). This suggests that fused dROb spheroids in mineralization media are capable of advancing toward bone maturation.

## 4 Discussion

In this project, osteogenic 3D macrotissues (2.64 ± 0.23 mm diameter) were engineered by guided fusion of dROb spheroids using the customized 3D bioassembly system. Modular tissue engineering is a bottom-up approach aimed at recreating biomimetic tissues at a macroscale level. Modular or microscale tissues such as spheroids, cell sheets, and cell-laden hydrogels can be used as building blocks to bioassemble into a macroscale tissue (22). In this study, spheroids were used as building blocks due to their close mimicry of natural tissue formation by self-assembly and self-organization. Despite recent developments in bioassembling techniques to produce larger tissues (13, 18, 23, 24), non-invasive and non-destructive bioassembly remains a challenge. Our study involved developing a simple bioassembly system (**Figure 1C**) using a novel non-invasive temporary pillar array support (**Figure 1B**) to fabricate bone macrotissues.

### 4.1 Characterization of dROb spheroids

Prior to the investigation into macrotissue formation, dRObs were assessed for their spheroid-forming ability and the effect of different seeding densities on cell proliferation and viability, cellular arrangement, and ECM production. dRObs at three seeding densities (1 × 10^4^, 5 × 10^4^, 1 × 10^5^ cells) required 12 h, 36 h, and 48 h, respectively, to form compactly aggregated spheroids (**Figure 2A**). This suggests that complete cell aggregation time is dependent on the seeding density; the lower the seeding density, the faster the spheroid formation. This is in agreement with other cell types such as 3 × 10^4^ iPSCs completely aggregating in 24 h (25) and 2.5 × 10^5^ MC3T3 cells aggregating in 2 days (26).

Under the influence of mineralization media, dROb spheroids went through a cell proliferation phase till day 14 which then ceased (**Figure 2C**). Calcium deposits observed from day 14 onwards confirmed that dRObs have entered the mineralization phase (**Figure 4C**). This is an interesting finding that cell proliferation ceased when mineralization began. Similar observations were reported in other studies during osteogenic differentiation of osteoblast-like cells (27) and human adipose mesenchymal stem cells (haMSCs) (28). Moreover, despite arrested cell proliferation, the spheroid diameter increased over time which is suggestive of increased extracellular calcium production (26). These observations demonstrate that dRObs undergo osteogenesis when cultured in mineralization media in all three seeding densities.

The necrotic core size increased over time with increasing spheroid size, regardless of seeding density (**Figure 4B**). This is associated with a reduction in cell viability over time confirming cell death in the core region (**Figure 2D**). The presence of a necrotic core has not been reported in other osteogenic spheroids (26, 29, 30) which may be due to cell type and size differences. These studies produced spheroids of approximately 600 µm; however, dRObs formed spheroids of >1 mm. Despite the presence of a necrotic core, dROb spheroids continued to grow and mineralize. Studies showed high levels of hypoxia-inducible factor 1-alpha (HIF-1α) during endochondral ossification, which suggests that a hypoxic microenvironment can support bone formation, i.e., promotes differentiation of osteoblasts to osteocytes (31, 32). Therefore, the necrosis observed in dROb spheroids might be beneficial for osteocytogenesis. This lays the ground for future investigation of osteocyte formation and characterization in dROb spheroids.

Based on the interest of using dROb spheroids that reach a larger size (~1.5 mm) relatively sooner (day 14), 1 × 10^5^ seeding density was considered ideal and used for depositing the spheroids in pillar array supports of current printed size (**Figure 1B**). However, a future study with lower seeding densities in downsized pillar array supports might be advantageous in reducing the necrotic core.

### 4.2 Three-dimensional macrotissue fabrication

Pillar arrays manufactured from Clear Resin^®^ (Formlabs) were used as temporary supports to hold spheroids in place (**Figure 5A**). Subsequent physical removal of the pillar array support would leave the tissue construct scaffold-free for maturation. With pillar array supports, we successfully demonstrated the fusion of mineralized dROb spheroids within 2 days of bioassembly (**Figure 5C**). In the literature, the Ozbolat research group bioassembled osteogenic spheroids using the sacrificial material made of alginate and calcium chloride which was sacrificed by citrate chelation (16, 24, 33, 34). Considering the possible detrimental effects of citrate on extracellular calcium, our pillar array scaffold method would be beneficial. Other approaches such as PEGT/PBT copolymer (13), PCL microwell arrays (35), Kenzan needle arrays (18), and self-healing support hydrogels (36) have also been used for the bioassembly of spheroids. However, there are some limitations in these approaches compared with the pillar array support method. For example, the permanent presence of scaffold materials PEGT/PBT and PCL (13, 35) might hinder mechanical signal transduction between cells (10). The Kenzan method is an invasive method involving needle insertion into spheroids which could be detrimental to cells (18). Support hydrogels take a longer time (4 days) for spheroid fusion (36) than pillar array supports (2 days) as well as there is a possibility of dilution and disturbance to the support hydrogels during media changes leading to loss of mechanical strength to hold spheroids. In addition, the inability to exchange media would affect the viability of metabolically demanding cells. Thus, pillar array supports are beneficial for rapid fusion of dROb spheroids without detrimental effects on spheroid integrity and extracellular calcium as well as for holding spheroids even under excessive manipulation. The method could be further enhanced by improving the ease of separation of macrotissues from the pillar following fusion, for example by using a non-fouling material such as poly(ethylene glycol) that cells would adhere less to or a sacrificial material that could be removed by a method less detrimental to mineralized tissues than citrate chelation.

After removal of the pillar array supports, the fusion between spheroids extended from the mere edges (on dAR2) to the close contact reorganization encompassing all spheroids together (on dAR4 and dAR8) making it a macrotissue of approximately 2.6 mm diameter (**Figure 6A**). Calcium deposits detected by staining (**Figure 6B**) and bone-like hydroxyapatite mineral structures in SEM imaging (**Figure 8F**) demonstrate that dROb macrotissues are capable of producing appropriate bone extracellular matrix. Additionally, the presence of collagen networks and compactly arranged cells through lamellipodia and filopodia shows cell–cell and cell–ECM interactions. This proves that our bioassembly approach is effective in maintaining the functionality of dROb cells to produce bone-specific mineralization in large-scaled tissues. Energy-dispersive X-ray analysis to quantify bone-specific hydroxyapatite mineral content was attempted which was not successful in quantifying phosphorus due to masking of phosphate peaks by osmium used during sample preparation. Further analysis is required after modifying the sample preparation procedure.

Relative Alizarin red quantification assay would provide information on macrotissues’ ability to continually increase ECM mineral synthesis during and after fusion. However, this assay is commonly used for 2D culture (37) and needs modification to extract minerals from core regions of tightly packed 3D macrotissues.

Runx2 gene downregulation and the presence of osteocalcin (late osteogenic marker) in dROb macrotissues (**Figures 9A, C**) reveal that the cells are in late osteogenic phase, i.e., mature osteoblasts and early osteocytes in association with hydroxyapatite deposition (38–40). Furthermore, upregulated ALP expression on dAR2 shows that the cells are undergoing matrix maturation. Although there is no significant difference, the decline of ALP expression on dAR8 might suggest its progress toward osteocyte predominance over osteoblasts (41, 42). These findings provide a base for future investigation to confirm the presence of osteocytes by extending the culture period of macrotissues. Osteocyte-specific immuno-markers like podoplanin (43) and significantly reduced ALP expression over time (41) would confirm the presence of osteocytes.

Based on these findings from single-layered bioassembly, multilayered bioassembly of dROb spheroids can be investigated for further scaling up of bone tissue constructs. A potential limitation of the multilayered bioassembly approach is that the spheroids were transferred individually which would be time-consuming during further upscaling of tissue. Also, the necrotic core in multilayered bioassembly is important to be considered as larger tissue areas in the core would be deprived of oxygen and nutrients.

Overall, dROb macrotissue developed by our novel bioassembly system can be a viable 3D *in-vitro* model of bone tissue. Rat-originated osteoblast cells were used in this study due to their easy availability and close biological resemblance to human cells (44). We predict that this bioassembly setup could be used as a novel methodology to engineer a variety of other types of macrotissues such as tendon, muscle, or multitissue constructs using cells of human origin.

## 5 Conclusion

In this study, we fabricated a 3D *in-vitro* bone macrotissue model using differentiated rat osteoblasts which recapitulate the mineralization of native bone tissue. The bioassembly approach using a temporary pillar array support is simple and effective in manufacturing a scaffold-free macrotissue product without any physical and/or chemical damage. This fabricated model and bioassembly system can be widely used in tissue engineering and pharmacological research to understand bone-related diseases and their treatment strategies.

## Data availability statement

The original contributions presented in the study are included in the article/supplementary materials, further inquiries can be directed to the corresponding author/s.

## Author contributions

VP: Conceptualization, Data curation, Formal analysis, Funding acquisition, Investigation, Methodology, Validation, Visualization, Writing – original draft, Writing – review & editing. FM: Conceptualization, Funding acquisition, Methodology, Resources, Supervision, Writing – review & editing. LM: Methodology, Resources, Supervision, Writing – review & editing. JP: Conceptualization, Funding acquisition, Methodology, Project administration, Resources, Supervision, Writing – review & editing.

## References

[1] el Demellawy D Davila J Shaw A Nasr Y. Brief review on metabolic bone disease. Acad Forensic Pathol (2018) 8:611–40. doi: 10.1177/1925362118797737 PMC6490580 31240061

[2] Drake MT Cremers S Russell RG Bilezikian JP. Drugs for the treatment of metabolic bone diseases. Br J Clin Pharmacol (2019) 85:1049–51. doi: 10.1111/bcp.13857 PMC6533457 30950086

[3] Skjødt MK Frost M Abrahamsen B. Side effects of drugs for osteoporosis and metastatic bone disease. Br J Clin Pharmacol (2019) 85:1063–71. doi: 10.1111/bcp.13759 PMC6533454 30192026

[4] Stubenrouch FE Cohen ES Bossuyt PMM Koelemay MJW van der Vet PCR Ubbink DT. Systematic review of reporting benefits and harms of surgical interventions in randomized clinical trials. BJS Open (2020) 4:171–81. doi: 10.1002/bjs5.50240 PMC7093777 32207574

[5] Amini AR Laurencin CT Nukavarapu SP. Bone tissue engineering: recent advances and challenges. Crit Rev BioMed Eng (2012) 40:363–408. doi: 10.1615/CritRevBiomedEng.v40.i5.10 23339648 PMC3766369

[6] de Wildt BWM Ansari S Sommerdijk NAJM Ito K Akiva A Hofmann S. From bone regeneration to three-dimensional in *vitro* models: tissue engineering of organized bone extracellular matrix. Curr Opin BioMed Eng (2019) 10:107–15. doi: 10.1016/j.cobme.2019.05.005

[7] Laurencin CT Ambrosio AM Borden MD Cooper JA. Tissue engineering: orthopedic applications. Annu Rev BioMed Eng (1999) 1:19–46. doi: 10.1146/annurev.bioeng.1.1.19 11701481

[8] Yuste I Luciano FC González-Burgos E Lalatsa A Serrano DR. Mimicking bone microenvironment: 2D and 3D in *vitro* models of human osteoblasts. Pharmacol Res (2021) 169:105626. doi: 10.1016/j.phrs.2021.105626 33892092

[9] Lin X Patil S Gao Y-G Qian A. The bone extracellular matrix in bone formation and regeneration. Front Pharmacol (2020) 11:757. doi: 10.3389/fphar.2020.00757 32528290 PMC7264100

[10] Athanasiou KA Eswaramoorthy R Hadidi P Hu JC. Self-organization and the self-assembling process in tissue engineering. Annu Rev BioMed Eng (2013) 15:115–36. doi: 10.1146/annurev-bioeng-071812-152423 PMC4420200 23701238

[11] DuRaine GD Brown WE Hu JC Athanasiou KA. Emergence of scaffold-free approaches for tissue engineering musculoskeletal cartilages. Ann BioMed Eng (2015) 43:543–54. doi: 10.1007/s10439-014-1161-y PMC4380596 25331099

[12] Decarli MC Amaral R dos Santos DP Tofani LB Katayama E Rezende RA. Cell spheroids as a versatile research platform: formation mechanisms, high throughput production, characterization and applications. Biofabrication (2021) 13:032002. doi: 10.1088/1758-5090/abe6f2 33592595

[13] Mekhileri NV Lim KS Brown GCJ Mutreja I Schon BS Hooper GJ. Automated 3D bioassembly of micro-tissues for biofabrication of hybrid tissue engineered constructs. Biofabrication (2018) 10:024103. doi: 10.1088/1758-5090/aa9ef1 29199637

[14] Mironov V Visconti RP Kasyanov V Forgacs G Drake CJ Markwald RR. Organ printing: tissue spheroids as building blocks. Biomaterials (2009) 30:2164–74. doi: 10.1016/j.biomaterials.2008.12.084 PMC3773699 19176247

[15] Tripathi S Mandal SS Bauri S Maiti P. 3D bioprinting and its innovative approach for biomedical applications. MedComm (2023) 4(1):e194. doi: 10.1002/mco2.194 36582305 PMC9790048

[16] Heo DN Ayan B Dey M Banerjee D Wee H Lewis GS. Aspiration-assisted bioprinting of co-cultured osteogenic spheroids for bone tissue engineering. Biofabrication (2021) 13:015013. doi: 10.1088/1758-5090/abc1bf 33059343

[17] Itoh M Nakayama K Noguchi R Kamohara K Furukawa K Uchihashi K. Scaffold-free tubular tissues created by a bio-3D printer undergo remodeling and endothelialization when implanted in rat aortae. PloS One (2015) 10:e0136681. doi: 10.1371/journal.pone.0136681 26325298 PMC4556622

[18] Aguilar IN Smith LJ Olivos DJ Chu T-MG Kacena MA Wagner DR. Scaffold-free bioprinting of mesenchymal stem cells with the regenova printer: optimization of printing parameters. Bioprinting (Amsterdam Netherlands) (2019) 15:e00048. doi: 10.1016/j.bprint.2019.e00048 31457110 PMC6711201

[19] Livak KJ Schmittgen TD. Analysis of relative gene expression data using real-time quantitative PCR and the 2(-Delta Delta C(T)) Method. Methods (2001) 25:402–8. doi: 10.1006/meth.2001.1262 11846609

[20] Hammer Ø Harper DAT Ryan PD. Past: Paleontological statistics software package for education and data analysis. Palaeontol Electron (2001) 4:178.

[21] Elmore S. Apoptosis: a review of programmed cell death. Toxicol Pathol (2007) 35:495–516. doi: 10.1080/01926230701320337 17562483 PMC2117903

[22] Nichol JW Khademhosseini A. Modular tissue engineering: engineering biological tissues from the bottom up. Soft Matter (2009) 5:1312–9. doi: 10.1039/b814285h PMC2826124 20179781

[23] Lindberg GCJ Cui X Durham M Veenendaal L Schon BS Hooper GJ. Probing multicellular tissue fusion of cocultured spheroids-A 3D-bioassembly model. Adv Sci (Weinheim Baden-Wurttemberg Ger (2021) 8:e2103320. doi: 10.1002/advs.202103320 PMC8596109 34632729

[24] Kim MH Banerjee D Celik N Ozbolat IT. Aspiration-assisted freeform bioprinting of mesenchymal stem cell spheroids within alginate microgels. Biofabrication (2022) 14:024103. doi: 10.1088/1758-5090/ac4dd8 PMC8855887 35062000

[25] Zhang M Shi J Xie M Wen J Niibe K Zhang X. Recapitulation of cartilage/bone formation using iPSCs via biomimetic 3D rotary culture approach for developmental engineering. Biomaterials (2020) 260:120334. doi: 10.1016/j.biomaterials.2020.120334 32862124

[26] Koblenzer M Weiler M Fragoulis A Rütten S Pufe T Jahr H. Physiological mineralization during *in vitro* osteogenesis in a biomimetic spheroid culture model. Cells (2022) 11:2702. doi: 10.3390/cells11172702 36078105 PMC9454617

[27] Gentili C Bianco P Neri M Malpeli M Campanile G Castagnola P. Cell proliferation, extracellular matrix mineralization, and ovotransferrin transient expression during in *vitro* differentiation of chick hypertrophic chondrocytes into osteoblast-like cells. J Cell Biol (1993) 122:703–12. doi: 10.1083/jcb.122.3.703 PMC2119661 8393014

[28] Hanna H Mir LM Andre FM. *In vitro* osteoblastic differentiation of mesenchymal stem cells generates cell layers with distinct properties. Stem Cell Res Ther (2018) 9:203. doi: 10.1186/s13287-018-0942-x 30053888 PMC6063016

[29] Ayan B Wu Y Karuppagounder V Kamal F Ozbolat IT. Aspiration-assisted bioprinting of the osteochondral interface. Sci Rep (2020) 10:13148. doi: 10.1038/s41598-020-69960-6 32753630 PMC7403300

[30] Wolff A Frank M Staehlke S Springer A Hahn O Meyer J. 3D spheroid cultivation alters the extent and progression of osteogenic differentiation of mesenchymal stem/stromal cells compared to 2D cultivation. Biomedicines (2023) 11:1049. doi: 10.3390/biomedicines11041049 37189667 PMC10135665

[31] Amarilio R Viukov SV Sharir A Eshkar-Oren I Johnson RS Zelzer E. HIF1alpha regulation of Sox9 is necessary to maintain differentiation of hypoxic prechondrogenic cells during early skeletogenesis. Development (2007) 134:3917–28. doi: 10.1242/dev.008441 17913788

[32] Kim J Adachi T. Cell condensation triggers the differentiation of osteoblast precursor cells to osteocyte-like cells. Front Bioeng Biotechnol (2019) 7:288. doi: 10.3389/fbioe.2019.00288 31709248 PMC6819367

[33] Ayan B Celik N Zhang Z Zhou K Kim MH Banerjee D. Aspiration-assisted freeform bioprinting of prefabricated tissue spheroids in a yield-stress gel. Commun Phys (2020) 3:183. doi: 10.1038/s42005-020-00449-4 33251340 PMC7695349

[34] Akkouch A Yu Y Ozbolat IT. Microfabrication of scaffold-free tissue strands for three-dimensional tissue engineering. Biofabrication (2015) 7:31002. doi: 10.1088/1758-5090/7/3/031002 26373778

[35] Burdis R Chariyev-Prinz F Kelly DJ. Bioprinting of biomimetic self-organised cartilage with a supporting joint fixation device. Biofabrication (2021) 14:015008. doi: 10.1088/1758-5090/ac36be 34825656

[36] Daly AC Davidson MD Burdick JA. 3D bioprinting of high cell-density heterogeneous tissue models through spheroid fusion within self-healing hydrogels. Nat Commun (2021) 12:753. doi: 10.1038/s41467-021-21029-2 33531489 PMC7854667

[37] Gregory CA Gunn WG Peister A Prockop DJ. An Alizarin red-based assay of mineralization by adherent cells in culture: comparison with cetylpyridinium chloride extraction. Anal Biochem (2004) 329:77–84. doi: 10.1016/J.AB.2004.02.002 15136169

[38] Owen TA Aronow M Shalhoub V Barone LM Wilming L Tassinari MS. Progressive development of the rat osteoblast phenotype in *vitro*: reciprocal relationships in expression of genes associated with osteoblast proliferation and differentiation during formation of the bone extracellular matrix. J Cell Physiol (1990) 143:420–30. doi: 10.1002/jcp.1041430304 1694181

[39] Mukherjee S Sharma S Soni V Joshi A Gaikwad A Bellare J. Improved osteoblast function on titanium implant surfaces coated with nanocomposite Apatite-Wollastonite-Chitosan- an experimental *in-vitro* study. J Mater Sci Mater Med (2022) 33:25. doi: 10.1007/s10856-022-06651-w 35190908 PMC8860945

[40] Komori T. Regulation of proliferation, differentiation and functions of osteoblasts by runx2. Int J Mol Sci (2019) 20:1694. doi: 10.3390/ijms20071694 30987410 PMC6480215

[41] Kato Y Windle JJ Koop BA Mundy GR Bonewald LF. Establishment of an osteocyte-like cell line, MLO-Y4. J Bone Miner Res (1997) 12:2014–23. doi: 10.1359/jbmr.1997.12.12.2014 9421234

[42] Amarasekara DS Kim S Rho J. Regulation of osteoblast differentiation by cytokine networks. Int J Mol Sci (2021) 22:2851. doi: 10.3390/ijms22062851 33799644 PMC7998677

[43] Kaur K Das S Ghosh S. Regulation of human osteoblast-to-osteocyte differentiation by direct-write 3D microperiodic hydroxyapatite scaffolds. ACS Omega (2019) 4:1504–15. doi: 10.1021/acsomega.8b03272

[44] Czekanska EM Stoddart MJ Richards RG Hayes JS. In search of an osteoblast cell model for in *vitro* research. Eur Cells Mater (2012) 24:1–17. doi: 10.22203/ECM.V024A01 22777949

